# Limb Perfusion Delivery of a rAAV1 Alpha-1 Antitrypsin Vector in Non-Human Primates Is Safe but Insufficient for Therapy

**DOI:** 10.3390/genes15091188

**Published:** 2024-09-10

**Authors:** Debora Pires-Ferreira, Darcy Reil, Qiushi Tang, Meghan Blackwood, Thomas Gallagher, Allison M. Keeler, Jessica A. Chichester, Kristin K. Vyhnal, Jane A. Lindborg, Janet Benson, Dongtao Fu, Terence R. Flotte, Alisha M. Gruntman

**Affiliations:** 1 Horae Gene Therapy Center, University of Massachusetts Chan Medical School, Worcester, MA 01605, USA; debora.piresferreira@umassmed.edu (D.P.-F.); darcy.reil@umassmed.edu (D.R.); qiushi.tang@umassmed.edu (Q.T.); meghan.blackwood@umassmed.edu (M.B.); thomas.gallagher@umassmed.edu (T.G.); allison.keeler@umassmed.edu (A.M.K.);; 2 Department of Pediatrics, University of Massachusetts Chan Medical School, Worcester, MA 01605, USA; 3 NeuroNexus Institute, University of Massachusetts Chan Medical School, Worcester, MA 01605, USA; 4 Gene Therapy Program, Department of Medicine, Perelman School of Medicine, University of Pennsylvania, Philadelphia, PA 19104, USA; 5 Lovelace Biomedical Research Institute, Albuquerque, NM 87108, USA; kvynhal@lovelacebiomedical.org (K.K.V.); jlindborg@lovelacebiomedical.org (J.A.L.); jbenson@lovelacebiomedical.org (J.B.); 6 Department of Pathology, Immunology, and Laboratory Medicine, University of Florida, Gainesville, FL 32610, USA; fudo@pathology.ufl.edu

**Keywords:** alpha-1 anti-trypsin, AATD gene therapy, rAAV gene therapy, rAAV safety, biodistribution, pre-clinical study

## Abstract

Background/Objectives: α-1 antitrypsin (AAT) deficiency is an inherited, genetic condition characterized by reduced serum levels of AAT and increased risk of developing emphysema and liver disease. AAT is normally synthesized primarily in the liver, but muscle-targeting with a recombinant adeno-associated virus (rAAV) vector for α-1 antitrypsin (AAT) gene therapy has been used to minimize liver exposure to the virus and hepatotoxicity. Clinical trials of direct intramuscular (IM) administration of rAAV1-hAAT have demonstrated its overall safety and transgene expression for 5 years. However, the failure to reach the therapeutic target level after 100 large-volume (1.5 mL) IM injections of maximally concentrated vector led us to pursue a muscle-targeting approach using isolated limb perfusion. This targets the rAAV to a greater muscle mass and allows for a higher total volume (and thereby a higher dose) than is tolerable by multiple direct IM injections. Limb perfusion has been shown to be feasible in non-human primates using the rAAV1 serotype and a ubiquitous promoter expressing an epitope-tagged AAT matched to the host species. Methods: In this study, we performed a biodistribution and preclinical safety study in non-human primates with a clinical candidate rAAV1-human AAT (hAAT) vector at doses ranging from 3.0 × 10^12^ to 1.3 × 10^13^ vg/kg, bracketing those used in our clinical trials. Results: We found that limb perfusion delivery of rAAV1-hAAT was safe and showed a biodistribution pattern similar to previous studies. However, serum levels of AAT obtained with high-dose limb perfusion still reached only ~50% of the target serum levels. Conclusions: Our results suggest that clinically effective AAT gene therapy may ultimately require delivery at doses between 3.5 × 10^13^–1 × 10^14^ vg/kg, which is within the dose range used for approved rAAV gene therapies. Muscle-targeting strategies could be incorporated when delivering systemic administration of high-dose rAAV gene therapies to increase transduction of muscle tissues and reduce the burden on the liver, especially in diseases that can present with hepatotoxicity such as AAT deficiency.

## 1. Introduction

Recombinant adeno-associated virus (rAAV)-based gene therapy has emerged as a promising therapeutic choice for treating a wide range of genetic conditions. The therapeutic efficacy of AAV vectors has been confirmed by the successful treatment of life-threatening conditions such as hemophilia A and B [1,2]. However, contrary to initial assumptions that there would be minimal immune responses to rAAV vectors, reports of adverse events involving immune responses directed against the vector capsid or the transgene product have emerged as clinical translation has advanced, posing significant limitations to therapeutic efficacy and patient safety [3]. The intricate interplay between vector-induced immune responses and associated toxic effects highlights the necessity for exploring alternative dosing strategies and administration routes to mitigate associated risks.

α-1 antitrypsin deficiency (AATD) is a genetic disorder characterized by a deficiency in the production of α-1 antitrypsin (AAT), a protease inhibitor primarily synthesized in the liver. This deficiency leads to impaired regulation of neutrophil elastase activity, resulting in the destruction of lung tissue and subsequent development of emphysema, particularly in individuals with a history of smoking [4,5]. Additionally, AATD can lead to hepatotoxicity due to the accumulation of abnormal AAT protein aggregates within hepatocytes. These aggregates disrupt normal liver function, leading to cellular injury, inflammation, and potential progression to liver fibrosis, cirrhosis, or hepatocellular carcinoma [6]. Early studies of AAT gene therapy were able to achieve stable expression following a single vector injection into the muscle, and higher expression could be achieved by delivering the vector via portal vein injection [7,8]. However, two previous studies, one in non-human primates (NHPs) and one in mice, have shown that intravenous (IV) rAAV-AAT can exacerbate underlying liver disease [9,10].

Therefore, AATD gene therapy strategies that spare the liver are preferred to avoid worsening the existing hepatic stress, particularly when using a high vector dosage [11]. Elevated liver enzymes (such as serum alanine aminotransferase [ALT] and/or aspartate aminotransferase [AST]) are markers for liver inflammation which are often associated with reduced expression of the transgene and rising of circulating AAV-specific CD8 T cells post-vector administration. The capsid is rapidly cleared in the host in a dose-dependent manner which has been partially explained through accelerated hepatocyte turnover and increased antigen presentation due to systemic dosing. Microscopic alterations consistent with liver damage have been identified after systemic rAAV delivery, further indicating liver damage [12]. Moreover, in a recent trial targeting X-linked myotubular myopathy, a condition affecting both muscles and the liver, four participants died weeks after undergoing high-dose IV rAAV gene transfer therapy (1–3 × 10^14^ vector genomes [vg]/kg) due to liver failure. Administration of immunosuppressive medication did not produce any improvement and examination of the patients’ livers revealed no evidence of cellular infiltration. This led to the conclusion that the fatal liver damage was likely a result of toxicity from the vector or the transgene product rather than being triggered by an immune response mediated by T cells [13]. Thus, there is a need for liver-sparing approaches to deliver gene therapies.

In contrast with systemic administration, intramuscular (IM) injection has been demonstrated to retain the rAAV vector within a tissue for an extended period of time, facilitating sustained expression of the transgene. Clinical trials conducted by our research team have established the safety and efficacy of delivering rAAV1-CB-hAAT to the muscles of AATD patients without immunosuppression. These trials demonstrated a dose–response relationship (ranging from 6.0 × 10^11^ to 6.0 × 10^12^ vg/kg), achieving a maximum patient AAT serum level of approximately 300 nM (2–3% of therapeutic levels [11 μM]). We found that the serum levels of AAT were stable for up to 5 years following a single set of 100 IM injections, which indicates the potential for long-term effectiveness [14,15]. However, further dose escalation is limited because of technical challenges in concentrating the vector and the increased pain caused by patients receiving additional IM injections [12,14,15]. The persistent AAT protein expression in serum was observed despite elevated serum creatine kinase (CK) levels and the detection of IFNγ-secreting cells to AAV1 capsid in peripheral blood mononuclear cells (PBMCs). This persistence was attributed to the presence of muscle-infiltrated capsid-specific regulatory T cells (Tregs) and exhausted T cells, which were observed as soon as day 21 following dosing and lasted for up to 5 years following vector delivery, along with rAAV capsid persistence in situ [12]. These findings were observed in both NHPs and humans, suggesting that elevated inflammatory enzymes in the serum may not necessarily indicate a detrimental immune response causing loss of transgene expression [12,14,15,16].

In order to increase delivered dose and gene expression while preserving safety, locoregional delivery strategies for AAV vectors have been developed [17,18,19]. A subsequent study investigated regional limb perfusion strategies in NHPs and found that rAAV1 administered (6.0 × 10^12^ vg/kg) via venous limb perfusion (VLP) doubled AAT serum levels compared to direct IM injection and showed superior vector genome retention in the muscle (25-fold increase) while maintaining a favorable safety profile [20]. In another study, a rAAV8 vector expressing the highly immunogenic green fluorescent protein (GFP [7 ×10^12^ vg/kg]) was administered to cynomolgus monkeys via limb perfusion, and GFP expression was sustained for up to 1 year despite a peripheral immune response to the transgene. Interestingly, the persistent inflammation observed was accompanied by resident Treg cells, suggesting that limb perfusion administration enhances long-term transgenic expression by promoting the generation of in situ Treg cells [16]. These findings indicate that limb perfusion delivery is a promising approach for increasing dosage scalability in gene replacement therapy.

In this study, we employed the limb perfusion method to administer the rAAV1-human AAT (hAAT) vector into the skeletal muscle of cynomolgus macaques at doses ranging from 3.0 × 10^12^ to 1.2 × 10^13^ vg/kg (bracketing doses used in our clinical trials) and assessed biodistribution and preclinical safety. Our findings indicated that while this approach facilitated safe transgenic expression, serum levels of AAT at the highest dose were not sufficient to reach the therapeutic threshold. The data generated here and in our previous NHP study [10] suggest that local IV delivery protocols at a dosage of 1 × 10^14^ vg/kg is necessary to attain clinically effective gene therapy for AAT deficiency.

## 2. Materials and Methods

### 2.1. Animals

Cynomolgus macaques (*Macaca fascicularis*) were purchased from Worldwide Primates (Miami, FL, USA). Animals were pre-screened for neutralizing antibodies to AAV1 and only those with acceptably low titers were purchased for study. All animal study procedures were approved by the Lovelace Biomedical Institutional Animal Care and Use Committee. The animals were quarantined and housed according to the Standard Operating Procedures consistent with the Office of Laboratory Animal Welfare Guide for the Care and Use of Laboratory Animals [21].

The animals were 1.5 to 4 years old and weighed 2.5–5 kg. Animals were uniquely identified by numerical tattoos applied by the vendor. All animals received methylprednisolone acetate orally for immune suppression. The initial dose was 1 mg/kg from study days 0 to 30, and gradually reduced to 0.125 mg/kg every other day after study day 44.

### 2.2. AAV Vector Constructs, Production, and Purification

**Figure 1:**
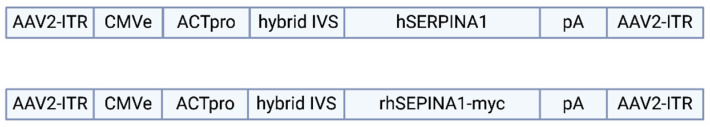
Maps of AAV vector constructs. Linear maps of the AAV gene cassettes used for this study. AAV2-ITR = inverted terminal repeat sequence from AAV2, CMVe = CMV immediate early enhancer, ACTpro = chicken β-actin promoter, Hybrid IVS = hybrid intron with upstream portion from chicken β-actin and downstream portion from rabbit β-globin, hSERPINA1= human *SERPINA1* coding sequence, rhSERPINA1-myc = rhesus *SERPINA1* coding sequence with c-myc epitope tag fusion, pA = SV40 polyadenylation signal.

The vector DNA cassette used for these studies is the same vector cassette that was previously used in three clinical trials. It was packaged in AAV2 capsids in one trial and AAV1 capsids in the other two. The cassette consists of a Cytomegalovirus (CMV) enhancer/chicken β-actin promoter/hybrid rabbit β-globin intron (an expression cassette known as CB or CAG) driving expression of the normal human *SERPINA1* gene (PI*M allele) followed by an SV40 polyadenylation signal and flanked by AAV2 inverted terminal repeats [14,22,23,24]. This construct (rAAV1-CB-hAAT) was packaged in AAV1 capsids and purified using previously published techniques [25,26]. In addition, a second vector with the same design expressing a myc-tagged version of the rhesus *SERPINA1* was also packaged in AAV1 capsids using the same methods (Figure 1) [10].

### 2.3. Vector and Vehicle Control

The test articles rAAV1-CB-hAAT (lot number YA-014.2) and rAAV1-CB-rhAAT-myc (Lot number L14-05-004) were produced at Applied Genetics Technology Corporation, Alachua, FL. Vector characteristics including identity, lot number, concentration, AAV infectious titer, sterility, and purity (endotoxin and mycoplasma contamination) were determined. Lactated Ringer’s Injection (lot number 575872) was purchased from ICU Medical (San Clemente, CA, USA).

### 2.4. Assignment of NHPs to Groups

Animals were divided into four groups with one male and one female in each group. Group 1 received a Ringer lactate solution as a vehicle control. Group 2 received the rAAV1-CB-hAAT vector at a dose of 3.0 × 10^12^ vg/kg. Group 3 received the rAAV1-CB-hAAT vector at a dose of 1.2 × 10^13^ vg/kg. Group 4 received the rAAV1-CB-rhAATmyc vector at a dose of 6.0 × 10^12^ vg/kg.

### 2.5. Experimental Design

Animals received the vehicle or TA formulation via hydrodynamic peripheral vein infusion (HPV) administration on study day 0 as shown in Table 1. Animals were monitored for clinical signs and body weight throughout the study. Blood was collected at various occasions to analyze hematology, serum chemistry, neutralizing antibodies to AAV 1, and AAT expression. Urinalysis was performed at baseline and on study days 3 and 7. Muscle biopsy was performed on study day 28 for immunohistochemical (IHC) analysis. Animals were euthanized on study day 90 and complete necropsies were performed. Tissue samples were collected for histology, immunohistochemistry, and biodistribution of vector and transgene expression.

### 2.6. Neutralizing Antibody Response to rAAV1 Vector

Serum from NHPs was collected pre-dosing and at day 90 post-dosing, serial diluted (starting at dilutions of 1:5 for pre-dosing samples and at 1:20 for post-dosing samples, then subsequently diluted 1:2 several times), and then heat-inactivated for 30 min at 56 °C. Samples were incubated with AAV1-CMV-LacZ viral particles (1 × 10^9^ gc/well) for 1 h at 37 °C in a 5% CO_2_ environment to allow for neutralizing antibodies (NAbs) in the serum samples to bind to the AAV1 viral particles. This mixture was then added to Huh7 cells (1 × 10^5^ cells) which were previously infected with adenovirus (100 particles/well) and incubated for 18–22 h at 37 °C in a 5% CO_2_ environment. Cells were washed and developed with a Galactor-Star kit (Applied Biosystems, Waltham, MA, USA) and luminescence was measured with a luminometer to measure LacZ activity. The neutralizing antibody (NAb) titers are expressed as the highest dilution that inhibited β-galactosidase expression by at least 50% compared to a negative mouse serum control (Table 1).

### 2.7. Intravascular Limb Infusion

The locoregional IV perfusion protocol was adapted from Su et al. [27]. Briefly, the vector was diluted in lactated Ringer’s solution. The volume injected was approximately 20% of the limb volume, which was estimated to be 12 mL/kg. A cannulation was placed in the saphenous vein with a catheter and a tourniquet was placed around the proximal pelvic extremity and inflated to a pressure of 300 mmHg. The injection was started after 10 min of an ischemic period and performed over 5 min. The tourniquet was released after 20 min. Unfortunately, one animal in Group 3 (3002) had the vector delivered without having the torniquet applied, which effectively caused the delivery to be intravenous rather than limb perfusion. This animal was replaced with another (3003). However, the dose that was delivered to this animal partially leaked out of the vein when being administered.

### 2.8. Clinical and Detailed Observations

Clinical observations were performed twice daily (morning and afternoon). Observations were oriented towards (1) identifying dead, weak, or moribund animals, and (2) documenting the onset and progression of any abnormal clinical signs. Detailed clinical observations were performed at baseline and on days 0 (post-dosing), 1–7 (2× daily), 13, 20, 27, 28 (post-biopsy), 29–35 (2× daily), and then weekly until necropsy. Animals were weighed weekly until scheduled euthanasia.

### 2.9. Vector Biodistribution

The vector biodistribution was analyzed from a biopsy of the rectus femoris muscle collected at post-delivery day 28 and in tissues harvested at day 90 during necropsy. The tissues were immediately frozen upon being collected. The analysis of genomic material was performed by digital droplet PCR (ddPCR) using a primer/probe targeting the miR-resistant region within the plasmid and the cynomolgus albumin gene as a reference. Vector biodistribution was measured in the liver, spleen, lung, heart, kidney, gonads, brain, injection site, lymph node, and various muscles.

### 2.10. Blood Sample Collection, Preparation, and Analysis

#### 2.10.1. Clinical Pathology

Blood samples were collected for the following analyses: hematology, 1× K_3_EDTA tube (minimum of 0.5 mL); clinical chemistry, 1× clot/SST tube (up to 2.0 mL). Following collection, all samples were inverted to ensure proper mixing. Hematology samples were analyzed by automated analyses using an ADVIA™2120 Hematology System Analyzer (Siemens Medical Solutions, Malvern, PA, USA). The parameters for hematology are shown in Supplementary Table S1. A blood smear was prepared and analyzed microscopically for slide verification. For clinical chemistry analyses, whole blood was centrifuged and separated into cellular and serum fractions. Serum samples were analyzed at LBRI on a Hitachi cobas c501Chemistry Analyzer (Roche Diagnostics, Indianapolis, IN, USA) or a Vitros 5600 Integrated Chemistry System (Ortho Clinical Diagnostics, Rochester, NY, USA). The clinical chemistry parameters to be measured or calculated are shown in Supplementary Table S2.

#### 2.10.2. Urine Collection for Clinical Pathology Analysis

Urine was collected at baseline, and on study days 3 and 7. Urine volume, color, and appearance were recorded. Glucose, bilirubin, urobilinogen, ketone, protein, presence of blood, and pH were assessed by DipStick (Siemens Multistix [Siemens Medical Solutions] or equivalent). Microscopic examinations were conducted to determine the presence of mucus, crystals, WBC, RBC, epithelial cells, and bacteria.

### 2.11. AAT Expression

#### 2.11.1. Human AAT ELISA

High-binding extra 96-well plates (Immulon 4; Dynatech Laboratories, Chantilly, VA, USA) were coated with 100 mL of human-specific goat anti-AAT (1:2000 dilution; Bethyl Laboratories, Montgomery, TX, USA) in Voller’s buffer (Na_2_CO_3_, NaHCO_3_, NaN_3_, and dH_2_O, adjusted to a pH of 9.6) overnight at 4 °C. After blocking with 1% nonfat dry milk in phosphate-buffered saline (PBS) with Tween 20 (PBST), duplicate standard curves (Athens Research and Technology, Athens, GA, USA) and serially diluted experimental samples were incubated in the plate for 1 h at room temperature (RT), then a second goat anti-hAAT (horseradish peroxidase) antibody (1:5000 dilution; Bethyl Laboratories) was incubated in the plate at RT for 1 h. The plates were washed with PBST between reactions. After reaction with 3,3′, 5,5′ tetramethylbenzidine dihydrochloride peroxidase substrate (KPL, Gaithersburg, MD, USA), reactions were stopped by adding 2 N H_2_SO_4_ (Fisher Scientific, Hudson, NH, USA). Plates were read at 450 nm on a VersaMax microplate reader (Molecular Devices, Sunnyvale, CA, USA).

#### 2.11.2. c-myc-AAT ELISA

The c-myc ELISA protocol was performed as described for the human AAT ELISA with the following changes: the plates were coated with an anti-c-myc (1:1000 dilution; Bethyl Laboratories) antibody and the plates were blocked with 3% bovine serum albumin.

#### 2.11.3. c-myc-AAT Western Blot

NHP serum samples were diluted 1:50 in PBS. A series of c-myc solutions were prepared at concentrations ranging from 500 to 2500 ng/mL to make a standard curve for quantifying protein concentrations. Both the serum samples and c-myc standards were added to sample loading buffer and then subjected to denaturation at 95 °C for 5 min. Samples were centrifuged at 12,000 rpm for 5 min to pellet any debris and ensure homogeneity. Samples were then separated by sodium dodecyl-sulfate polyacrylamide gel electrophoresis (SDS-PAGE), transferred to nitrocellulose membranes, and blocked in Odyssey blocking solution for 1 h at RT. The membranes were then incubated with a primary c-myc antibody diluted 1:1000 in Odyssey blocking solution with Tween 20 followed by a secondary fluorophore-conjugated antibody diluted 1:5000 in Odyssey blocking solution with Tween 20. Membranes were imaged using an Odyssey LiCOR Imaging System (LI-COR Biotech, Lincoln, NE, USA).

#### 2.11.4. Human AAT Immunohistochemistry

Immunohistochemistry was carried out by the Molecular Pathology Core at the University of Florida. Briefly, the IHC stain was performed on a Leica Bond RXm Autostainer (Leica Biosystems, Deer Park, IL, USA). The 4 μm serial sections were de-paraffinized with Bond Dewax Solution (Leica Biosystems) and rehydrated with water, including an intermediary step to quench endogenous peroxidase activity (peroxidase block). Slides were rinsed in 1× IHC wash buffer (Leica Biosystems) and then co-incubated with an anti-AAT (1:1000, Fitzgerald, Acton, MA, USA) antibody for 30 min. Slides were rinsed in 1× IHC wash Buffer followed by incubation with NovoLink Polymer (Leica Biosystems) for 8 min at RT. Detection of AAT was achieved by incubating slides in 3’3’ diaminobenzidine (Leica Biosystems) for 10 min at RT. Slides were counterstained with hematoxylin (Leica Biosystems, Deer Park, IL, USA) for 5 min and mounted with Cytoseal XYL (Richard-Allen Scientific, Kalamazoo, MI, USA). Human AAT (hAAT) expressed in muscle tissues appears as brown staining. Quantification was carried out using the QuPath v0.5.1 software for Windows. The results are presented as the percentage of area positive for hAAT (stained) relative to the overall area of the muscle tissue section.

## 3. Results

### 3.1. NHPs Groups and AAV1 NAbs

Animals received a low or high dose of the rAAV1-CB-hAAT vector (3.0 × 10^12^ or 1.2 × 10^13^ vg/kg) or the rAAV1-CB-rhAATmyc vector (6.0 × 10^12^ vg/kg). NAb titers in the serum increased in all groups following vector limb perfusion delivery of the rAAV1 vector compared to the vehicle control 90 days after dosing, indicating that the viral vector elicited a clear immunological response (Table 1). This suggests that circulating rAAV1 particles are likely to be neutralized by the host immune system and re-dosing to further boost AAT expression would need to incorporate immunomodulation strategies.

### 3.2. Safety Profile

All animals survived until scheduled necropsy. There were no abnormal clinical observations throughout the study. Body weights within dose groups did not change appreciably throughout the study. The majority of differences between control animals and TA-treated animals for the hematology, serum chemistry, and urinalysis endpoints at specific time points post-dosing were of minimal magnitude (<2-fold).

Detailed examinations were performed and consisted of a complete external and internal examination. Few abnormal gross findings were noted at scheduled necropsy, which included minimal to marked fibrous adhesions on the liver/diaphragm of several animals. In general, organ weights were unremarkable and microscopic examination of liver from all animals was within normal limits, and clinical pathology markers of potential muscle or liver damage were not consistently correlated with treatment.

Administration of rAAV1-CB-hAAT at the low dose (3.0 × 10^12^ vg/kg) by venous limb perfusion resulted in minor mononuclear cell infiltrates in some muscles of the hindlimb in both male and female animals, while the high dose (1.20 × 10^13^ vg/kg) resulted in minor mononuclear cell infiltrates which were occasionally accompanied by skeletal muscle degeneration in some muscles of the dosed hindlimb of the single male animal (a second male dosed via simple IV administration showed no muscular changes of the limb). The single female that received the high dose by venous limb perfusion showed more consistent and severe muscular changes including mononuclear cell inflammation, muscle degeneration, and muscle regeneration.

Administration of rAAV1-CB-rhAAT-myc at 6 × 10^12^ vg/kg did not result in consistent muscle or other changes, with the single male animal demonstrating minimal mononuclear infiltrates in one skeletal muscle in the non-dosed hindlimb (the relationship to treatment is unknown). Although relatively high levels of various liver and muscle related enzymes/markers (i.e., CK) occurred in most animals at one or more of the time points examined, we did not observe a pattern in the changes when examined across genders, study days, related parameters, doses, correlating histopathology, or vectors. These data were quite variable, and without consistent evidence of substantive changes which could be confidently related to administration of either test article at any time point examined.

The safety assessment was favorable, as no adverse effects were observed and the levels of liver enzymes (ALT, AST) and a muscle injury marker (CK) were normal. Additionally, the analysis of blood parameters (including platelet and white blood cell counts) found no discrepancies among the groups, nor any consistent deviations from the expected range, indicating a positive outcome of the safety assessments (Supplementary Figure S1).

### 3.3. CK

With respect to individual animals, the majority of differences between vehicle- and TA-treated animals at specific time points post-dosing between were small (<2-fold), and we did not observe sustained CK elevation.

### 3.4. Troponin

Serum samples were collected for determination of Troponin C Type 2 (TNNC2) concentration using ELISA. All samples, regardless of dose group and time point, were below the limit of detection.

### 3.5. IFNγ ELISpot

Blood samples were shipped at ambient temperature on the day of the procedure for processing at University of Pennsylvania for vector for analysis as previously described [28]. Peripheral blood mononuclear cells (PBMCs) were isolated from blood for all dose groups for the IFN-γ ELISpot assay at baseline, SD14, 30, 60, and at necropsy. The ELISpot assay demonstrated that five out of seven (71%) animals administered either rAAV1-CB-hAAT or rAAV1-CB-hAAT-myc generated positive IFN-γ responses following vector administration. Of these responders, two of them responded to the hA1AT peptide library only, one responded to the AAV1 capsid peptide library only, and two of them responded to both the capsid and transgene peptide libraries. Responses to the AAV1 capsid remained low in magnitude (<200 SFU per million cells) and were detected between study days 30 and 90. Responses to the hA1AT peptide library were low in magnitude in the low-dose rAAV1-CB-hAAT and the rAAV1-CB-hAAT-myc animals but did reach a moderate level (200–500 SFU per million cells) in the high-dose rAAV1-CB-hAAT group, which may be suggestive of a vector dose response. Two vector-dosed animals remained negative for IFN-γ production throughout the study as did the two vehicle control animals.

### 3.6. Biodistribution

**Figure 2:**
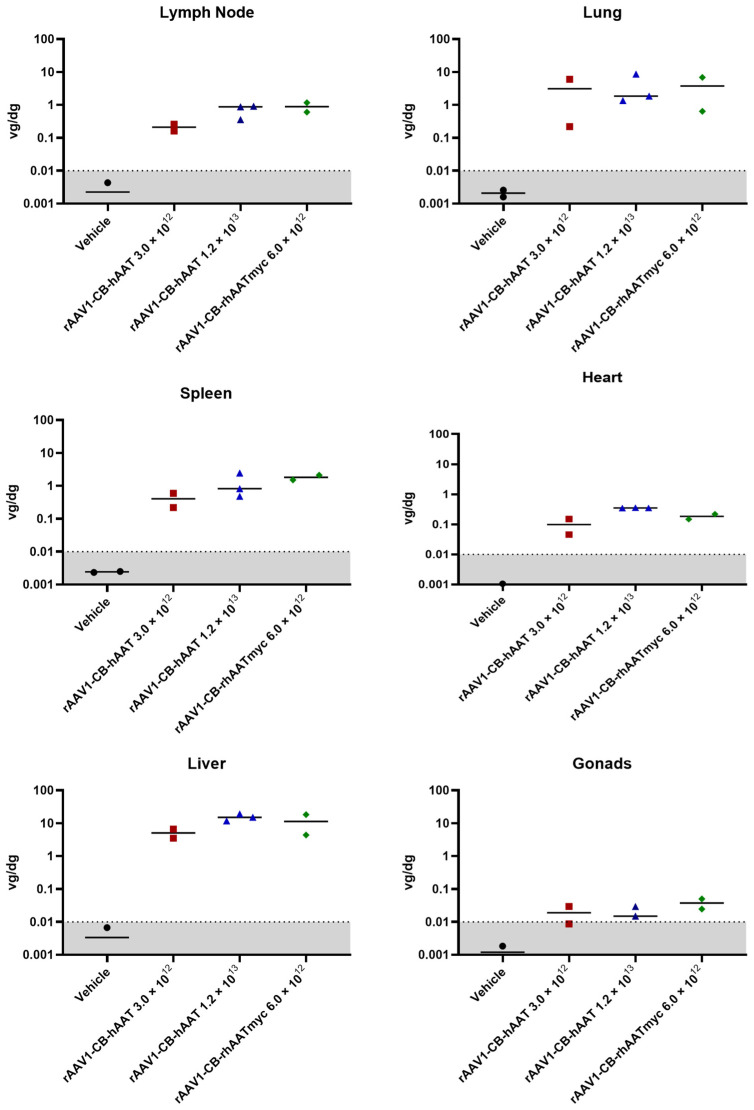
Vector genome biodistribution at injection site and muscles. Vector biodistribution was measured in the rectus femoris muscle taken from a biopsy at post-injection day 28 and at the injection site as well as in ipsilateral and contralateral gastrocnemius and rectus femoris muscles at post-injection day 90 from tissue samples collected during necropsy. Results are expressed as genome copies per diploid genome. Individual results are shown and lines indicate means.

**Figure 3:**
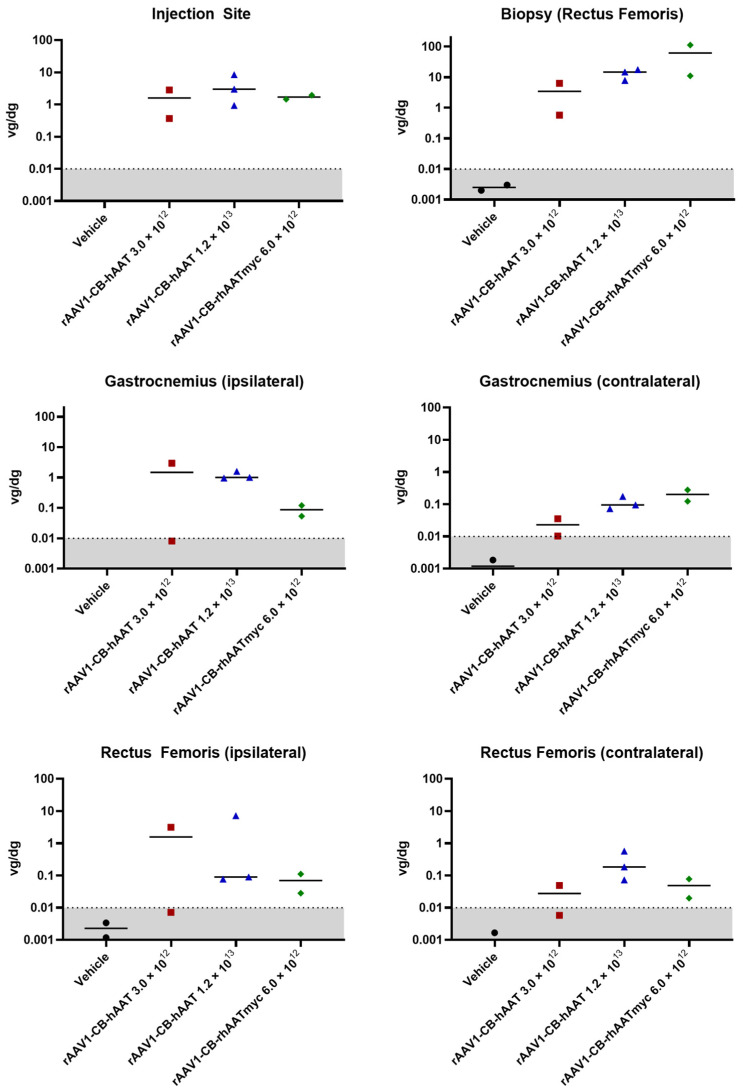
Vector genome biodistribution in tissues. Vector biodistribution was measured in the lymph node, lung, spleen, heart, liver, and gonads at post-injection day 90 from tissue samples collected during necropsy. Results are expressed as the number of vector genome copies per diploid genome. Individual results are shown and lines indicate means.

A skeletal muscle biopsy was obtained on day 28 and various tissues were harvested during necropsy at day 90 post limb perfusion delivery, and vector copy number was analyzed by ddPCR. Our findings indicate that the rAAV1 vector genomes were retained in the skeletal muscle for at least 90 days following limb perfusion administration, with the greatest abundance at the biopsy site, but significant delivery to both ipsilateral and contralateral limbs (Figure 2). We detected the vector genome in the lymph node, lung, spleen, heart, and liver, but minimal levels were found in the gonads (Figure 3). The biodistribution patterns of rAAV1-CB-hAAT and rAAV1-CB-rhAATmyc vectors were similar with the exception of the heart, where the high-dose rAAV1-CB-hAAT (1.2 × 10^13^ vg/kg) resulted in a higher number of genome copies compared to the low-dose (3.0 × 10^12^ vg/kg) and the rAAV1-CB-rhAATmyc vector.

### 3.7. AAT Gene Expression

#### Transgene Expression

**Figure 4:**
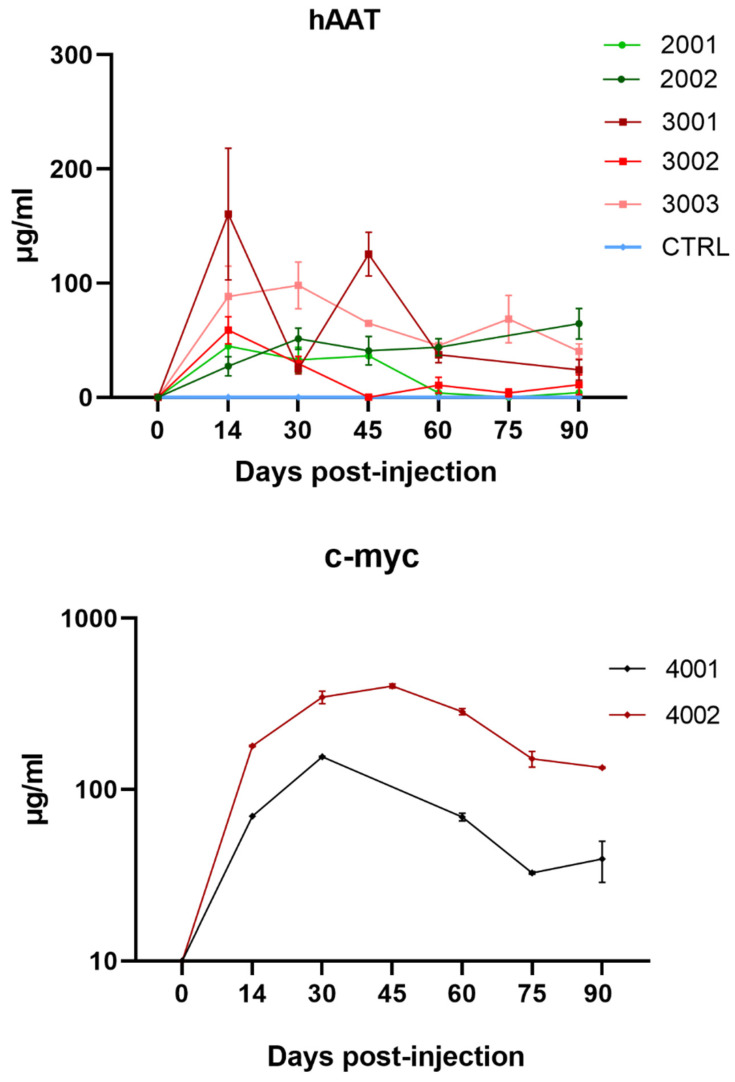
AAT serum levels. ELISA assays were performed on serum samples from NHPs that received the vector carrying the human AAT gene (rAAV1-CB-hAAT), the c-myc-AAT gene (rAAV1-CB-rhAATmyc), or vehicle control. Expression levels of hAAT or c-myc were quantified from serum samples collected prior to vector injection (day 0) and at 14, 30, 45, 60, 75, and 90 days post-injection. Data are shown as mean ± standard error of the mean. Two technical replicates were performed of the hAAT samples and four technical replicates were performed of the c-myc samples.

Both high-dose rAAV1-CB-hAAT (1.2 × 10^13^ vg/kg) and rAAV1-CB-rhAATmyc (6.0 × 10^12^ vg/kg) vectors induced significant serum AAT expression (~100–300 μg/mL), corresponding to ~50% of the therapeutic threshold (570 μg/mL), and persisted for up to 90 days after limb perfusion injection, as shown by both ELISA and Western blot analyses (Figure 4 and Figure S2). In contrast, low-dose rAAV1-CB-hAAT (3.0 × 10^12^ vg/kg) induced 2–6-fold lower AAT expression (~50 μg/mL), achieving only ~8% of the therapeutic threshold.

**Figure 5:**
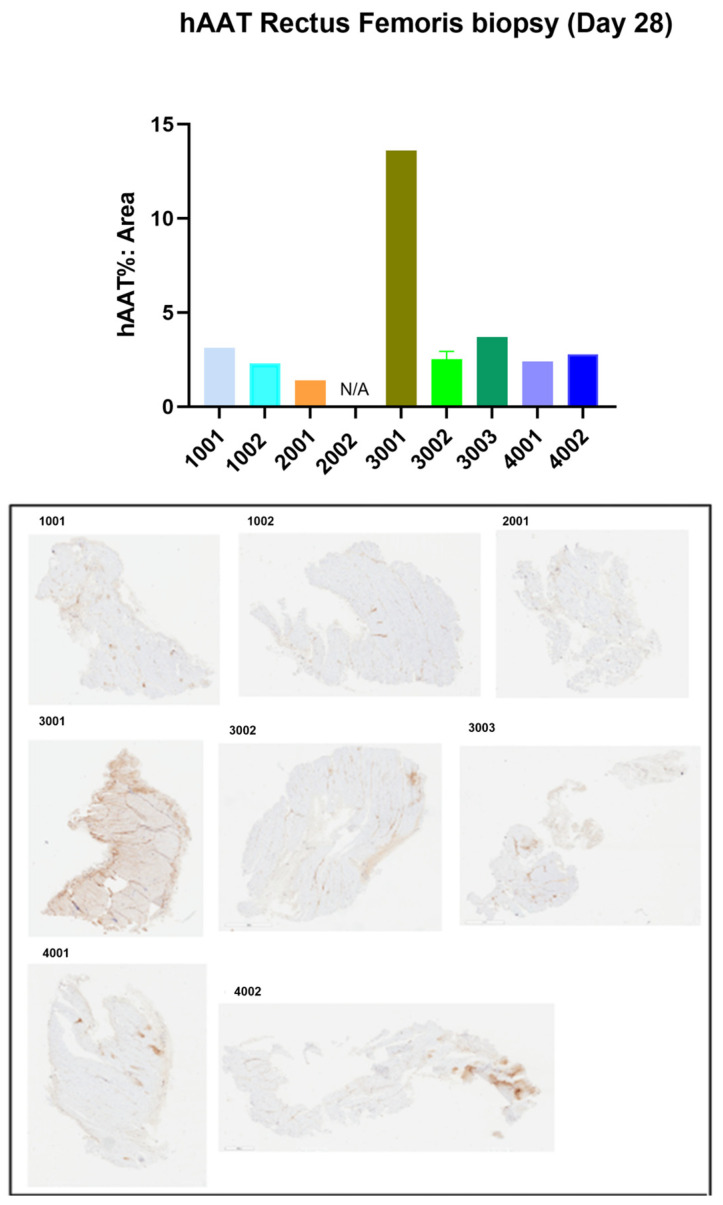
Immunohistochemical staining of muscle biopsy tissue. Skeletal muscle was collected by biopsy on day 28 post-injection, sections were immunohistochemically stained for hAAT, and the positive staining was quantified as a percentage of the total area. Tissue sections and quantification results are shown. Samples from animal 2002 were not able to be processed and the result is indicated as N/A. Three tissue sections from animal 3002 (mean ± standard error of the mean are shown) and one tissue section from the other animals were analyzed.

**Figure 6:**
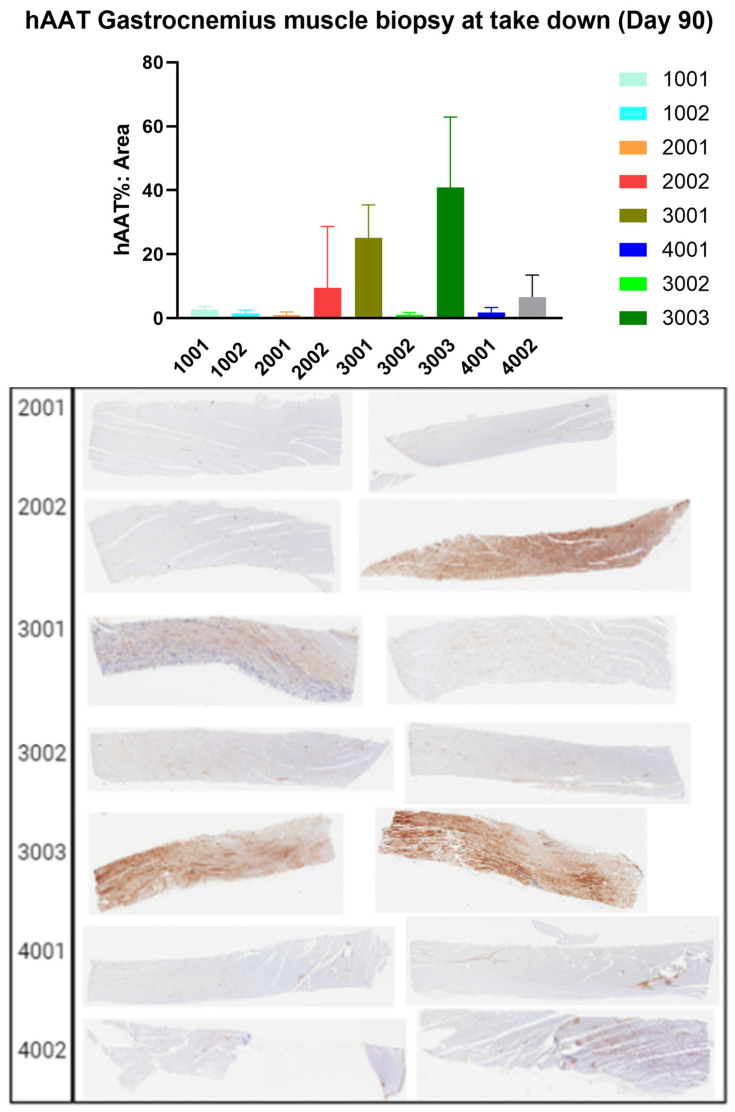
Immunohistochemical staining of muscle tissue sections. Skeletal muscle tissue was collected during necropsy at day 90 post-injections, sections were stained immunohistochemically stained for hAAT, and the positive staining was quantified as a percentage of the total area. Representative tissue sections and quantification results are shown. Two tissue sections were analyzed from each animal.

Expression of hAAT was assessed in muscle tissue by immunohistochemistry, which also indicated that the high dosages of rAAV1-CB-hAAT and rAAV1-CB-rhAATmyc resulted in greater expression of the therapeutic gene. We noticed widespread expression of hAAT in the muscle biopsy from one animal (3001) on day 28 (Figure 5). We noted some background staining of our antibody against hAAT, which we attribute to the similarity between human and cynomolgus macaque AAT proteins resulting in cross-reactivity of the antibody. At the conclusion of the study 90 days post-injection, tissue sections of skeletal muscle showed widespread expression of hAAT (Figure 6). However, we noted high variability in staining between serial sections taken from the same sample, indicating that expression of the vector appeared to be concentrated in areas of the muscle tissue. No observable alterations of the muscle tissue were found by histological examination.

## 4. Discussion

In the current study, we aimed to bridge between multiple IM injections and a limb perfusion approach and found the safety and biodistribution of limb perfusion rAAV1-CB-hAAT and rAAV1-CB-rhAATmyc delivery to NHPs to be favorable at doses ranging from 3.0 × 10^12^ to 1.2 × 10^13^ vg/kg, but still failed to reach the therapeutic serum threshold, achieving approximately 50% of the target level with the highest dose tested. The limb perfusion technique did not completely prevent biodistribution of vector genomes to the liver, with vector DNA detected at levels of 10 copies per diploid genome at the highest dose. However, this level of vector exposure was well-tolerated, unlike studies of IV administration in the same species of NHPs [29]. This route of administration is limited by the total vector volume that can be delivered. Therefore, limb perfusion could be an initial stage of delivery, which then transitions to systemic IV administration to achieve clinical success for rAAV-AAT gene therapies.

As the clinical experience rAAV gene has grown, liver toxicity has proven to be relatively common, particularly for systemic IV administration at doses above 3 × 10^13^ vg/kg [30,31,32,33]. Even at lower doses, IV administration of rAAV is frequently associated with hepatotoxicity in cases where effector T cell responses develop against AAV capsid proteins [34,35], although this is often manageable with oral corticosteroid treatment. The consequences of hepatotoxicity have been greater in diseases with pre-existing liver dysfunction, such as in X-linked myotubular myopathy [36]. This poses a significant concern for systemic AAV gene therapy in patients with AATD, as the disease leads to clinical hepatotoxicity in a subset of patients and subclinical liver dysfunction in many others [37]. This is challenging because a relatively high concentration of AAT in the serum (11 µM or 570 μg/mL) has generally been accepted as the therapeutic target for protein augmentation or gene therapy [37].

Direct IM injections of rAAV1-hAAT at doses ranging from 6.0 × 10^11^–6.0 × 10^12^ have previously been used in humans to avoid liver pathology and resulted in safe and sustained expression of serum AAT in patients for up to 5 years. However, the gene delivery achieved by this approach, which involved delivery of 134 mL of vector in 100 separate IM injections, needs to be increased 30-fold to reach the therapeutic serum level [12,14,15]. As an alternative approach, our group has recently completed a dose-ranging, safety, and biodistribution study for IV administration of a “dual-function” vector. This vector was designed to express a synthetic miRNA to silence endogenous mutant AAT while simultaneously expressing a wild-type AAT gene containing synonymous base substitutions to make it resistant to the miRNA [10]. Unfortunately, this approach has also failed to achieve target therapeutic levels, as a dose of 2.5 × 10^13^ vg/kg was only able to achieve ~20% of the target expression level. This suggests that doses of 1 × 10^14^ vg/kg could be needed to achieve therapeutic efficacy, but this carries additional risk of causing treatment-related adverse effects such as hepatotoxicity. Limb perfusion has been pursued as a strategy to concentrate transduction of viral particles in the muscle tissue and employ these cells to produce AAT that will be secreted into the circulation rather than burdening hepatocytes. Going forward, further optimization of limb perfusion strategies and vector design specific to this approach could yield improved expression of AAT in the bloodstream. Alternatively, the best liver-sparing approach for AAT gene therapy may be one of several gene-, base-, or prime-editing approaches that are currently in development [38,39,40]. While such approaches may still make use of rAAV to deliver gene-editing machinery, these approaches rely on the selective survival and propagation advantages that genomically corrected hepatocytes have over those burdened by the mutation to selectively repopulate the liver over time [41]. This reduces the volume that needs to be delivered and targets the liver rather than avoiding it. Thus, these technologies appear to be the most likely to provide long-term clinical benefit for this relatively common genetic disease.

Our study demonstrates that limb perfusion of rAAV1-hAAT at doses of 3 × 10^12^–1.2 × 10^13^ vg/kg was safe and resulted in efficient expression of the AAT gene in NHPs. In contrast to IV administration studies, we observed no evidence of treatment-induced hepatotoxicity, which is a significant concern in diseases associated with liver injury. However, this route of administration only achieved ~50% of the serum AAT expression that is thought to be needed for therapeutic efficacy. While this is an improvement over previous gene therapy approaches, it still falls short of meeting patients’ needs.

These findings suggest that achieving clinically effective AAT gene therapy may necessitate systemic IV delivery at doses ranging from 3.5 × 10^13^–1 × 10^14^ vg/kg, which is within the dose range utilized in approved rAAV gene therapies. Modifying the route of administration with approaches such as limb perfusion can be incorporated into IV delivery to enrich gene delivery to muscle tissue for AAT production and reduce viral transduction of the liver.

## Figures and Tables

**Table 1:** Experimental groups and NAbs.

Group	Treatment	Dose (vg/kg)	Animal ID	Sex	NAbs	
					(Pre-Screening)	(Post-Screening)
1	Vehicle	0	1001	Male	1:5	<1:20
			1002	Female	1:5	<1:20
2	rAAV1-CB-hAAT	3.0 × 10^12^	2001	Male	1:5	1:2560
			2002	Female	1:5	1:5120
3	rAAV1-CB-hAAT	1.2 × 10^13^	3001	Female	1:5	1:81,920
			3002 *	Male	1:5	1:20,480
			3003 **	Male	1:5	1:2560
4	rAAV1-CB-rhAATmyc	6.0 × 10^12^	4001	Female	1:5	1:10,240
			4002	Male	1:5	1:1280

Animals were screened for NAbs to AAV1 and only NHPs presenting NAb titers up to 1:5 were selected in order to prevent neutralization of the AAV vector. After vector administration, the NAb increased in the serum for all groups. * Tourniquet was not applied before the dose was delivered, so the vector was delivered systemically rather than by limb perfusion. ** Animal 3003 was added to replace 3002, but the dose partially leaked when being delivered, so the dosage was less than what was intended.

## Data Availability

All experimental laboratory data are available from the corresponding author upon reasonable request. All NHP data from this study have been deposited in the National Gene Vector Biorepository (NGVB) and requests for these data should be made to the NGVB Toxicology Study Archive (https://ngvbcc.org/ToxReportsView, accessed on 9 September 2024).

## Supplementary Materials

The following supporting information can be downloaded at: https://www.mdpi.com/article/10.3390/genes15091188/s1, Figure S1: Time course of AST, ALT, and CK levels; Figure S2: Western blot of dilutions and time course of serum c-myc expression; Table S1: Hematology parameters measured; Table S2: Serum chemistry parameters measured.
