# Supplementary material for: Limb Perfusion Delivery of a rAAV1 Alpha-1 Antitrypsin Vector in Non-Human Primates Is Safe but Insufficient for Therapy
Source: Genes (Basel). 2024 Sep 10;15(9):1188. doi: 10.3390/genes15091188 (PMC11431094; doi:10.3390/genes15091188)
Supplement: Supplementary file 1 [file genes-3183643-supplementary.pdf › genes-3183643-supplementary.pdf]

Supplemental Table S1. Hematology Parameters

Parameter	Abbreviation	Units
Red Blood Cell Count	RBC	$10^6/\mu L$
Hemoglobin	HGB	g/dL
Hematocrit	HCT	%
Mean Corpuscular Volume	MCV	fL
Mean Corpuscular Hemoglobin Concentration	MCHC	g/dL
Mean Corpuscular Hemoglobin	MCH	pg
Platelet Count	PLT	$10^3/\mu L$
Mean Platelet Volume	MPV	fL
Reticulocytes (Absolute)	RETIC	$10^9/L$
Percent Reticulocytes (Relative)	RETIC	% RBC
Red Cell Distribution Width	RDW	%
White Blood Cell Count and Absolute Differential		
White Blood Cell Count	WBC	$10^3/\mu L$
Neutrophils	PMN	$10^3/\mu L$ (and %)
Lymphocytes	LYM	$10^3/\mu L$ (and %)
Monocytes	MONO	$10^3/\mu L$ (and %)
Eosinophils	EOS	$10^3/\mu L$ (and %)
Basophils	BASO	$10^3/\mu L$ (and %)
Large Unstained Cells	LUC	$10^3/\mu L$ (and %)

Supplemental Table S2. Serum Chemistry Parameters

Analyte	Abbreviation	Units
Alanine Aminotransferase (Alanine Transaminase)-Serum	ALT	IU/L
Albumin	ALB	g/dL
Alkaline Phosphatase	ALP	IU/L
Aspartate Aminotransferase (Aspartate Transaminase)-Serum	AST	IU/L
Bilirubin (Total)	BILI-T	mg/dL
Blood Urea Nitrogen	BUN	mg/dL
Calcium	CA	mg/dL
Chloride	CL-S	mmol/L
Cholesterol (Total)	CHOL	mg/dL
Creatinine	CRE-S	mg/dL
Creatine (Phospho)Kinase	CK (CPK)	IU/L
Glucose	GLU	mg/dL
Gamma Glutamyltransferase	GGT	IU/L
Lactate Dehydrogenase	LDH	IU/L
Myoglobin	MYO	IU/L
Phosphate (Phosphorus)	PHOS	mg/dL
Protein (Total)	TP	g/dL
Sodium	NA-S	mmol/L
Potassium	K-S	mmol/L
Triglycerides	TRIG	mg/dL
Calculated Parameters and Ratios		
Albumin/Globulin	A/G	None
Blood Urea Nitrogen/Creatinine	BUN/CRE	None
Globulin	GLOBN	g/dL

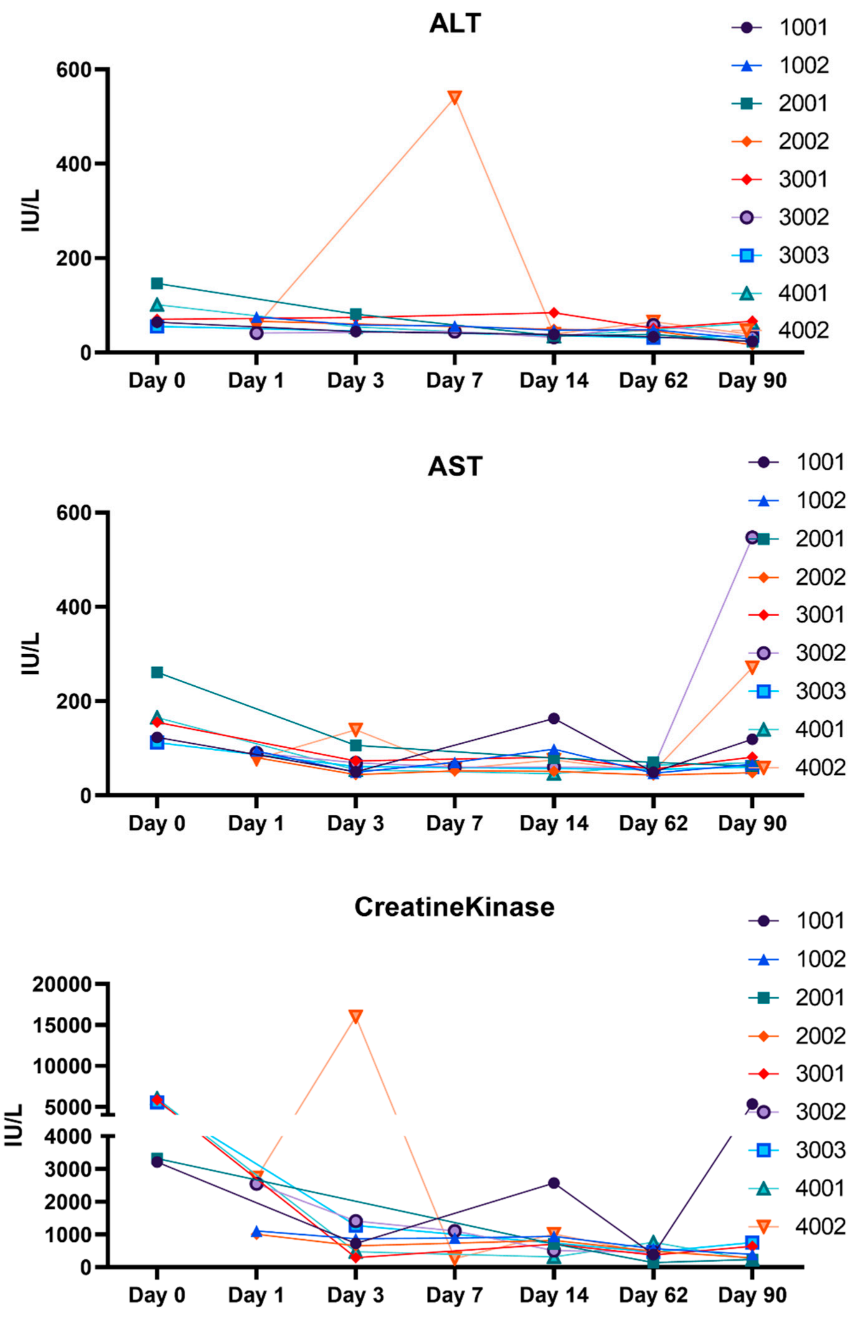

Figure S1. Time course of AST, ALT, and CK levels.

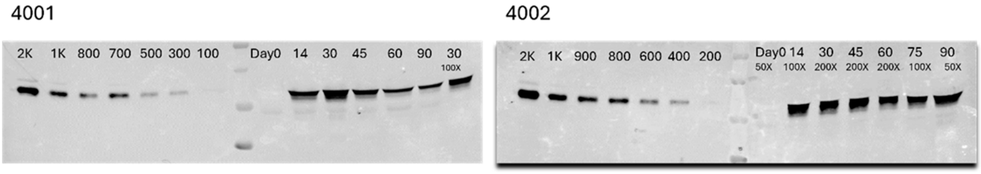

Figure S2: Western blot of dilutions and time course of serum c-myc expression
